# Prebiotic Effects of α- and β-Galactooligosaccharides: The Structure-Function Relation

**DOI:** 10.3390/molecules30040803

**Published:** 2025-02-09

**Authors:** Ina Ignatova, Alexander Arsov, Penka Petrova, Kaloyan Petrov

**Affiliations:** 1 Institute of Chemical Engineering, Bulgarian Academy of Sciences, 1113 Sofia, Bulgaria; ina.ignatova@iche.bas.bg; 2 Institute of Microbiology, Bulgarian Academy of Sciences, 1113 Sofia, Bulgaria; al.arsov@microbio.bas.bg (A.A.); ppetrova@microbio.bas.bg (P.P.)

**Keywords:** GOS, RFO, α-galactosidase, β-galactosidase, *Bifidobacterium*, *Lactobacillus*, probiotic

## Abstract

Oligosaccharides containing galactosyl moieties belong to two main groups: raffinose family oligosaccharides (RFO, α-GOS) and lactose-type β-galactooligosaccharides (β-GOS), both well-known for their prebiotic effect. The present review investigates the vast amounts of recent research on the structures of GOS and their beneficial impact. It focuses on the molecular interactions between GOS and probiotics in vitro and in vivo, the enzymology of the processes, and the genetic prerequisites for the synthesis and degradation of GOS by probiotic bacteria. The preferences of probiotic strains belonging to the *Bifidobacterium* and *Lactobacillus* genera are elucidated to form and degrade GOS of a certain length, structure, and linkages between monomers. A brief overview of the industrial production of β-GOS by natural and recombinant strains included the methods and production efficiency evaluation.

## 1. Introduction

The latest precise definition and scope of the term prebiotic was formulated in 2017 by the International Scientific Association for Probiotics and Prebiotics (ISAPP) as a “substrate that is selectively utilized by host microorganisms, providing a health benefit” [1]. The new criteria require a scientific demonstration that the food component or ingredient (i) can withstand the process of digestion, absorption, and adsorption of the host; (ii) is fermented by the microflora colonizing the gastrointestinal system; and (iii) selectively enhances the growth and/or activity of a limited or number of bacteria in the gastrointestinal system [2,3]. Thus, prebiotics are ingredients that are not destroyed in the gastrointestinal tract (GIT) but contribute to the growth of beneficial microorganisms and the associated improvement of immunity [4], resistance to pathogens [5], positive influence on metabolism [6], absorption of minerals [7], and mental health [8].

Because of the direct connection with probiotics, the global prebiotics market shows an expected annual growth rate of 14.9% from 2022 to 2030, reaching USD 21.2 billion by 2030. The global market for galactooligosaccharides (GOS) alone was estimated at USD 681.6 million in 2023 [9] and will probably reach USD 1.1 billion by 2030, growing at a compounded annual growth rate of 6.5% from 2023 to 2030 [9]. Clasado BioSciences (Berkshire, UK), Royal Friesland Campina (Amersfoort, The Netherlands), Yakult Honsha, Nissin Sugar Co. Ltd., and Megmilk Snow Brand Co., Ltd. (Tokyo, Japan), Ingredion (Westchester, IL, USA), First Milk Ltd. (Glasgow, UK), Kerry Group Plc. (Tralee, Ireland), Kowa Europe GmbH (Duesseldorf, Germany), and Samyang (Seoul, South Korea) hold the highest shares [10]. Europe and Japan dominate the market, with higher revenue due to the larger consumption of prebiotics by the elderly population and the development of countless infant formulas containing GOS.

Although a variety of food components claim a prebiotic nature, only two prebiotic types meet the criteria mentioned earlier, fructooligosaccharides (FOS) and GOS, as they have proven effects in human studies [11]. All others, like xylooligosaccharides, isomaltooligosaccharides, lactosucrose, mannanpectin, resistant starch, and oligodextrans, but also algae, fruit juices, peels, seeds, traditional Chinese medicines, polyphenols, and specific polypeptides remain among the “emerging prebiotics” until their functions in vivo are proven more convincingly [11,12].

**Figure 1:**
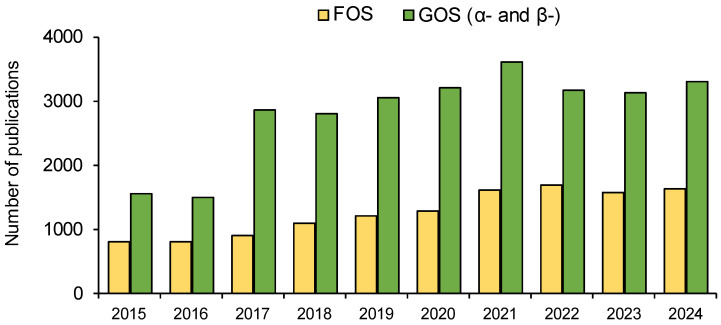
Scientific publications devoted to FOS and GOS prebiotics in the last ten years [14].

According to the American Association of Cereal Chemists, GOS and FOS are the dietary fiber with the highest impact due to their water solubility [13]. An average of 4100 scientific papers are published annually, dedicated to their production, degradation, and beneficial influence. Since GOS, compared to FOS, appears to be a prebiotic with greater structural diversity and has less studied effects on human health and many unknowns regarding its interactions with probiotic bacteria, over the last decade, there have been twice as many scientific articles describing GOS studies [14], as Figure 1 shows.

The main property of prebiotics is to directly or indirectly influence the health status. The positive effect of prebiotics is due to the synbiotic interactions with probiotic species colonizing the large intestine [15]. Long chains of indigestible carbohydrates stimulate the metabolism of bacteria in the peripheral parts of the digestive tract. Products of carbohydrate fermentation of GOS in the lower part of the GIT are short-chain fatty acids and lactic acid, which also determine the pH in the lumen [16,17]. There are three primary fatty acids: acetate, propionate, and butyrate, which are taken up by colonocytes and actively metabolized [18]. Various intestinal bacteria ferment GOS, including *Bifidobacterium*, *Bacteroides*, *Enterobacteriaceae,* and lactobacilli [19]. Increased abundance of *Bifidobacterium* spp. is the most commonly reported effect of GOS, and this is the so-called bifidogenic effect [20]. GOS also regulate the intestinal immune system, strengthen the intestinal barrier, inhibit the adhesion of pathogens, prevent infections and cancer, increase mineral absorption, and are included in specialized foods for lactose-intolerant individuals [21,22]. They prevent the adhesion of *Salmonella enterica* serovar *typhimurium* to murine enterocytes [23,24] and *Vibrio cholerae* and *Cronobacter sakazakii* to cell surface receptors on epithelial cells [25].

However, it has been shown that probiotic strains prefer different chain lengths, monomers, and specific linkages in prebiotic carbohydrates [26,27]. The literature reviews and experimental studies have shown that probiotic microbiota in functional foods synthesize β-GOS during fermentation, such as yogurt formation [28,29,30,31]. Still, other probiotic species also benefit from these prebiotics in the intestine. Therefore, beneficial food bacteria provide nutrients to those in the GIT, suggesting their connection in a ‘prebiotic carbohydrate cycle’.

On the other hand, α-galactosyl derivatives or raffinose (RFO, α-GOS) have not been sufficiently studied and probably have great potential as probiotics [32,33]. Bifidobacteria possess both α-galactosidase and cell-associated β-galactosidase activity, and α-GOS is a more efficient substrate for lactic acid production than FOS [34]. For example, while the probiotic *Lacticasebacillus casei* LAFTI L26 grows preferentially in inulin-containing media, for *Lactobacillus acidophilus* LAFTI L10, α-GOS is a preferred substrate. *Bifidobacterium animalis* ssp. *lactis* B94 also possesses the best growth at α-GOS [34].

Therefore, this review aims to elucidate the complex relationship between the prebiotic effects of GOS and their structure. This study summarizes the wide range of scientific works and their results accumulated over the last decade, focusing on the molecular mechanisms of GOS uptake by probiotic bacteria, the specificity of the active hydrolytic or transferase enzymes, and the results of the complex interaction in the invisible world of probiotics and prebiotics, from which human health ultimately benefits.

## 2. Chemical Structure of GOS, Natural Sources, and Industrial Synthesis

Galactans have a degree of polymerization (DP) varying from 2 to 10 monosaccharide units, according to the bonds (α- or β-) between the galactose units and the glucose. There are two main types of GOS: sucrose-related (raffinose-family oligosaccharides, RFO, α-GOS) and lactose-related (β-GOS). The chains typically end with a glucose or fructose residue; the remaining units are galactose [35]. The glycosidic bond impacts how these compounds are digested and utilized. The smallest molecules of the two types of GOS (melibiose, sucrose, and lactose) do not belong to the group by definition [36].

**Figure 2:**
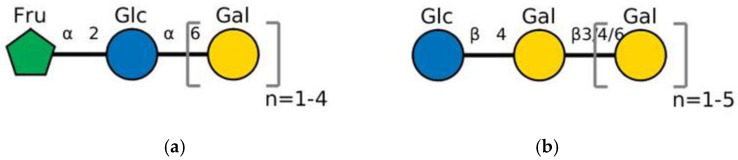
Prebiotic galactooligosaccharides composition. (**a**) α-GOS (RFO); (**b**) β-GOS. The typical α-GOS contain between one and four galactose moieties connected with α-(1→6) bonds; β-COS comprise lactose connected with one to five galactose residues linked with β-(1→3), β-(1→4), or β-(1→6) bonds. The structures were drawn using DrawGlycan-SNFGv2 software, available online at http://www.virtualglycome.org/DrawGlycan (accessed at 10 December 2024).

A schematic representation of the general structure of the two types of galactooligosaccharides is shown in Figure 2.

### 2.1. Raffinose Family Oligosaccharides (RFO, α-GOS)

RFO consist of linear chains of galactopyranosyl residues attached to the glucose moiety of sucrose via an α-(1→6)-galactopyranosidic linkage. According to Zu et al. [32], the “classical” RFO are elongated at the D-glucosyl residue of sucrose with α-(1→6) galactosyl residue to form stachyose with DP4 and verbascose with DP5, while in non-classical RFO, galactosyl extensions of raffinose are α-(1→1), α-(1→3), α-(1→4), or α-(1→6) linked, as well as being connected to a fructosyl group. α-GOS typically consist of three to six monosaccharide units. They are the second most abundant soluble carbohydrates in plants after sucrose [37]. RFO accumulate in tubers or other storage organs for the following stages of germination. Raffinose is the major oligosaccharide in most monocotyledon plants such as succulent epiphytes, major cereal grains (wheat, rice, barley, rye, oats, millet, sorghum, and maize), bamboo, bananas, ginger (*Zingiberales*) forage grasses, reeds and bromeliads (*Poales*), and seagrass. Its higher homologs, the tetrasaccharide stachyose and the pentasaccharide verbascose, accumulate in dicotyledon seeds, such as eudicots sunflower (*Helianthus*), dandelion (*Taraxacum*), cabbage (*Brassica*), apple (*Malus*), macadamia, and the whole family *Leguminosae*, commonly known as the legume, pea, or bean group [38]. Kotiguda et al. [39] identified the hexasaccharide ajugose in the roots and leaves of several *Lamiaceae*. The structures of the most studied α-GOS are shown in Table 1.

The functions of α-GOS in plants are storage reserves and cryoprotectants in frost-resistant plant organs [41]. They may play a role in desiccation tolerance and seed storage capacity acquisition. The content of RFO and other carbohydrates decreases with higher temperatures and increases during cold acclimatization in vegetative tissues [39].

### 2.2. Lactose Family Oligosaccharides (β-GOS)

β-GOS are carbohydrates composed of lactose and galactopyranosyl oligomers (DP 2-5) linked predominantly by β-(1,4) or β-(1,6) bonds, although low proportions of β-(1,2) and β-(1,3) linkages may also be present (Figure 2).

β-GOS are typically produced by enzymatic transgalactosylation of lactose by the enzyme β-galactosidase [42,43,44]. During enzymatic hydrolysis of lactose as the sole substrate, the liberated galactose is transferred to another lactose molecule [45]. Furthermore, while some GOS molecules possess a free anomeric carbon at the terminal end (making them reducing), others are non-reducing, instead containing a (1→1) linkage [46].

The chemical structures of two of the most widely used β-GOS, 3-galactosyl lactose and 4-galactosyl lactose, are shown in Table 2.

β-GOS are a common component of animal and human breast milk. In industrial production, they are synthesized from lactose as the primary raw material [47], using the enzyme β-galactosidase from different sources [35].

The enzyme source and reaction conditions possess an essential influence on both transgalactosylation and hydrolytic activities of β-galactosidases. β-Galactosidases in yeast and molds have wide industrial applications, the main advantages being their cost-effectiveness and thermal stability [47]. Their transgalactosylation products have been analyzed, and over 30 di-, tri-, and tetrasaccharides have been identified [45].

Because of the well-known safety, stability, high solubility, and neutral taste of β-GOS, they are ingredients of many food additives, especially infant formulas and functional foods [48,49].

## 3. Beneficial Effects of GOS Consumption

GOS are well-documented to promote the growth of beneficial gut bacteria, thus comprehensively influencing the intestinal microflora, including antibacterial effects, by preventing the adhesion of harmful microorganisms and modulating immune function [50,51,52]. Studies suggest that GOS can support gastrointestinal health, help regulate gut microbiota imbalances [38], alleviate ulcerative colitis [53], and positively impact certain neurological, metabolic, and allergic conditions [54,55]. They also influence metabolite production and essential aid in ion absorption and storage [56].

A commercial GOS mixture composed primarily of β-(1→4)-linked oligosaccharides showed an impressive ability to promote the growth of probiotic bacteria in a study that compared the effect of 10 different polysaccharides on 68 strains, 29 *Lactobacillus* and 39 *Bifidobacterium*. GOS were comparable to lactulose in the number of promoted strains and growth degree, as both were superior to inulin, maltodextrin, and polydextrose [57]. The same commercially available GOS—Vivinal^®^ (FrieslandCampina, Amersfoort, The Netherlands) reduce the cytotoxicity of enterohaemorrhagic *E. coli* (EHEC) by nearly 30%, more effectively than the probiotic strains *L. plantarum* CIDCA 83114 and *L. kefiri* CIDCA 8348 [58].

Prebiotics also promote the production of short-chain fatty acids (SCFAs) like acetate, propionate, and butyrate, which lower gut pH and inhibit acid-sensitive pathogens. Enterocytes can pass the SCFAs into blood circulation, so prebiotic compounds affect the gastrointestinal tract and other systems and organs [59,60]. Acetate aids in butyrate production, which is crucial for colonocyte health and gut lining maintenance [38]. A valuable biological function of acetate is its promotion of mineral absorption, particularly calcium, which is significant for bone health. Epidemiological research has documented the beneficial influence of propionate on allergic airway diseases. It indicates that as dietary fiber intake has declined, there has been a corresponding rise in allergy rates [54,55,61]. Butyrate significantly affects several functions within the colonic mucosa, including reducing inflammation and the risk of cancer, strengthening the defense barrier of the colon, and lowering oxidative stress. Additionally, butyrate may enhance the feeling of fullness [61]. Key mechanisms underlying these benefits are the inhibition of nuclear factor kappa B (NF-κB) activation and suppression of histone deacetylation [62]. Histone deacetylase inhibitors (HDACi) may reactivate tumor suppressor genes (TSGs), thus leading to a reduction in the tumor’s cell life [59,60].

The health benefits include immune system modulation, increased gut-specific immunoglobulins, regulatory interleukins, and reduced pro-inflammatory interleukins. According to some studies, newly synthesized and naturally occurring α-GOS demonstrated immunomodulatory properties by enhancing the secretion of nitric oxide (NO) and immune cytokines. Additionally, α-GOS with a higher DP showed greater effectiveness in regulating immune activity [43]. A study examined how a commercial prebiotic, Bimuno^®^ galactooligosaccharide B-GOS^®^ (Clasado BioSciences, Berkshire, UK), affected children with autism spectrum disorders (ASDs) over six weeks, focusing on gut health, behavior, and microbiota. Researchers found that children following exclusion diets (like gluten- or casein-free) experienced reduced gastrointestinal discomfort and showed notable behavioral improvements, particularly in social interactions. These benefits were more pronounced when prebiotic supplementation was included.

The prebiotic also significantly alters urinary metabolites, suggesting improved gut–brain communication. While gastrointestinal improvements were observed across the board, they were most impactful for those already on restrictive diets. The findings support that targeting gut health could enhance social behaviors and the overall well-being of children with ASD [55]. Palframan et al. [63] introduced the term prebiotic index (PI value) to assess the prebiotic effect quantitatively. It shows the ratio of beneficial and harmful bacteria in the microbial population grown at prebiotic substrates using the formula PI = (*Bifidobacterium*/Total) − (*Bacteroides*/Total) + (*Lactobacillus*/Total) − (*Clostridium*/Total). The conclusion was that the galactose-containing oligosaccharides were more effective prebiotics than the fructose-containing (FOS) and inulin since GOS have higher PI values. When tested at 1% w/v, at pH 6.8 for 24 h, β-GOS scored PI = 3.76, whereas FOS scored PI = 1.82. However, soybean oligosaccharides (RFO) obtained even higher PI scores of 4.36. Lactobacilli preferentially metabolize FOS, while bifidobacteria tend to prefer GOS. Additionally, the β-linkages in GOS, specifically the β(1→6) and β(1→3) bonds, showed greater prebiotic effectiveness and higher PI values compared to the β(1→4) linkages [64].

## 4. Enzyme Interactions Between Probiotic Strains and GOS

Structural considerations seem paramount for the prebiotic effect of GOS. A study investigated the ability of 13 commercial probiotic lactic acid bacteria (LAB) and bifidobacteria to digest 40 different GOS compounds from the commercial prebiotic Vivinal^®^. It turned out that *L. acidophilus* W37 and *B. infantis* DSM 20088 utilized 38 and 36 compounds, respectively, and had the broadest range of GOS used. *Bifidobacterium* spp. can digest even branched GOS with a higher DP, whereas most LAB strains use a more specific range of shorter GOS, preferring disaccharides [65].

Depending on the species, lactobacilli metabolize GOS via two distinct pathways operating with specific membrane transporters. More widespread is the import by lactose permease (LacS in *L. acidophilus*) and hydrolysis of the terminal galactose moieties by various β-galactosidases, for instance, LacZ (*L. acidophilus*) or LacLM (*Lactiplantibacillus plantarum*). The alternative pathway, less frequently employed, includes a PEP-dependent lactose phosphotransferase system (LacEF), which is responsible for the simultaneous import and phosphorylation; a phospho-β-galactosidase (LacG in *Lacticaseibacillus casei*) then hydrolyses the terminal β-galactose residue [66]. The predominance of the β-galactosidase pathway has been confirmed on the functional level. A study of 65 *Lactobacillus* strains from 15 different species revealed that most (41 strains) display considerably higher β-galactosidase than phospho-β-galactosidase activity. The exceptions are three strains of *Lacticaseibacillus casei*, two strains of *Lactococcus lactis*, and all 18 tested strains of *L. gasseri* in which β-galactosidase activity was virtually non-detectable. At the same time, the phospho-β-galactosidase activity was considerably higher than in most of the other strains used in the same study [35]. *Bifidobacterium* spp., among the first to colonize the infant gut and able to grow on a wide range of human milk oligosaccharides (HMOs), are a wealthy source of β-galactosidases. A comparative analysis of 11 β-galactosidases from *B. breve* UCC2003 and JCM 7017, *B. bifidum* LMG 13195, *B. longum* subsp. *infantis* 15697, and *B. longum* subsp. *longum* 8809 revealed significant variations in the range of substrates they could hydrolyze. The most versatile enzymes were able to utilize two different galactobioses (with ꞵ-(1→6) or ꞵ-(1→4) bonds) as well as lacto-*N*-tetraose and lacto-N-neotetraose. They belonged to the same Cluster 15 in all four subspecies, suggesting conserved β-galactosidase activities [67]. Two different β-galactosidases, Bga2A and Bga42A, able to degrade type-1 and type-2 HMOs (galactose linked to *N*-acetyl glucose via the ꞵ-(1→3) and ꞵ-(1→4) bonds, respectively), were isolated from *B. longum* ssp. *infantis* [68]. A metagenomic analysis of 16,000 clones led to the identification, cloning, and characterization of the novel β-galactosidase BgaC from *B. adolescentis*, an enzyme with low inhibition by galactose, high tolerance to glucose, and GOS-producing ability that makes it a promising candidate for the production of prebiotics in the dairy industry [69]. An extracellular β-1,4-galactanase from the GH53 family was identified in *Bacteroides thetaiotaomicron*, a prominent member of the human microbiota. This enzyme hydrolyzed GOS with DP3 and stimulated the growth of the probiotic *Lactobacillus* strains (*L. salivarius* W57, *Lacticaseibacillus paracasei* W20, and *Lacticaseibacillus casei* W56), which generally utilize GOS with DP2 primarily [70].

### 4.1. α-Galactosidases of Probiotic Bacteria

According to the mechanism of hydrolysis, glycoside hydrolases may be separated into “retaining” and “inverting”. Both mechanisms depend on two critical carboxylic amino acids in the active site. In retaining hydrolysis, one of the carboxylic amino acids acts as a nucleophilic group in forming the galactosyl-enzyme intermediate. In contrast, the other one acts as an acid-base catalyst and is responsible for activating the acceptor substrate. In inverting hydrolysis, the water molecule acts as a nucleophile, which attacks the anomeric carbon and causes a change in its configuration [71,72]. Inverting and retaining glycoside hydrolases may be distinguished by the distance between the critical carboxylic groups in the active site. The average retention distance for α- and β-glycosidases is 4.8 and 5.3 Å, respectively, while for inverting enzymes, it is larger, 9.0 and 9.5 Å for the α- and β-glycosidases, respectively [73]. α-Galactosidases (α-Gals, E.C. 3.2.1.22) are retention or inversion hydrolytic enzymes belonging primarily to six glycoside hydrolase (GH) families: GH4, GH27, GH36, GH57, GH97, and GH110. Bacterial enzymes belong to the GH4, GH36, GH57, and GH97 families, whereas GH27 contains eucaryotic enzymes (fungal, plant, and human). The glycosidic bonds attacked by α-Gals are usually α-(1→6) and are occasionally α-(1→3) with the enzymes that belong to GH27 and GH110. α-Galactosidase AgaBb of many *B. bifidum* strains, including the type strain ATCC 29521^TM^, belongs to GH110. The family GH97 contains both inverting and retaining α-Gals, as glutamate acts as a general base in inverting members, exemplified by *Bacteroides thetaiotaomicron* α-glucosidase BtGH97a, whereas an aspartate likely acts as a nucleophile in retaining members. Lee et al. reported that α-galactosidases and α-glucosidases belonging to GH97 share protein-fold similarities but have amino acid differences in the catalytic center. While glutamate is usually found as the catalytic residue in α-glucosidases, aspartic acids are common catalytically active amino acid residues in α-galactosidases. Unlike GH27 and GH36, a calcium-coordinated water molecule in the glutamate acid/base site is a characteristic of GH97 enzymes [74]. The primary substrates are melibiose, raffinose, stachyose, and verbascose (Table 3).

α-Gals from GH36 of 85 kDa are predominantly tetramers, although different oligomeric arrangements are reported for a few enzymes. The catalytic nucleophiles in the active site are usually aspartate and glutamate residues, with the notable exception of the GH4 enzymes, which require NAD+ and bivalent metal cations (Mn^2+^, Mg^2+^, Co^2+^, Ni^2+^, and Ca^2+^) as cofactors [78].

Aside from RFO, α-galactosidases can hydrolyze branched polysaccharides such as galacto- and glucomannans in legume seeds. Due to the transgalactosylation activity, α-galactosidases can form α-(1→3), α-(1→4), and α-(1→6) bonds between donors like melibiose and various acceptors as mono- and disaccharides, or polyols [79].

In humans, mutations in the gene for α-galactosidase A (GLA) lead to an X-linked, multisystemic, highly heterogenic, possibly life-threatening lysosomal storage disorder known as Fabry disease (FD) [80]. Oral administration of α-galactosidase A has alleviated the gastrointestinal syndromes of patients with FD [81]. The use of α-galactosidases as biopharmaceuticals extends well beyond enzyme replacement therapies for FD, for instance, to preventing xenorejection after transplantation by removing the α-Gal epitope, which may cause an acute immune response [82]. Even in the processing of agricultural waste and secondary agricultural process industries, α-galactosidases could find some application [83].

**Figure 3:**
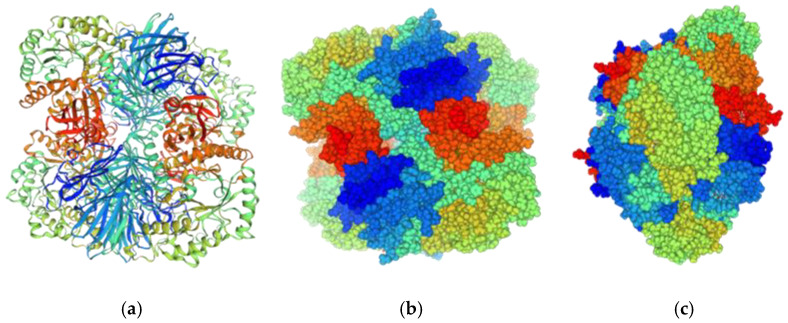
(**a**) Three-dimensional model of α-galactosidase of *L. acidophilus* NCFM of the GH36 family (tetramer) made in the SWISS-MODEL Workspace [85]; (**b**) space-filling model; (**c**) space-filling model rotated by 90° (vertical).

All probiotic bacterial strains of the genera *Bifidobacterium* and *Lactobacillus* contain genes encoding GH36 type I α-Gals organized in an operon that includes the *melA* gene and is induced in the presence of raffinose [84]. The α-galactosidase of *L. acidophilus* NCFM, a commercially produced probiotic, is also a tetramer belonging to the GH36 family. Its monomer MelA is composed of a large N-terminal twisted β-sandwich domain, connected by a long α-helix to the catalytic (β/α)8-barrel domain, with a C-terminal β-sheet domain. Four identical monomers form a tightly packed tetramer composed of four identical subunits, a relatively common arrangement in bacteria (Figure 3). Three monomers contributed to the structural integrity of each monomer’s active site. Moreover, the active site was much deeper than that of monomeric α-galactosidases (e.g., in *Thermotoga maritima*) from the same GH36 [84].

*Ruminococcus gnavus* E1, a human gut symbiont, produces a bifunctional enzyme with two catalytic domains, one involved in the hydrolysis of α-glycosides and an ATP-dependent domain that phosphorylates glucose. Kinetic studies suggest that this enzyme prefers short-chain RFO [86]. *B. bifidum* JCM 1254 is the source of *agabb*, a gene encoding α-galactosidase from GH110, a large protein of 1289 amino acids with several domains, including a carbohydrate-binding domain (CBM) 51, which can bind specifically to the blood group B antigen [87].

The *melA* locus in *Lpl. plantarum* ATCC 8014 is known to be clustered together with the β-galactosidase (*lacLM*) downstream and a putative galactoside transporter (*rafP*) upstream, showing high homology (>70%) with the LacS transporter from *Streptococcus thermophilus*. Both *melA* and *rafP* have their promoters and are transcribed as monocistronic mRNAs. The *melA* transcription was induced fourfold when the bacteria were grown with melibiose compared to glucose [88]. Promoter engineering can be used to express bacterial α-galactosidases in mammalian cells. This procedure, important for gene therapy and the production of transgenic animals, has been successfully tested in an intestine-derived cell line with a modified *lac* operon and the human mucin-2 promoter [89].

The discovery, purification, heterologous expression, and genetic engineering of α-galactosidases can potentially lead to dramatic changes in nutrition and lifestyle. They are crucial to the development of functional foods, with the ability to reduce anti-nutritional compounds. To enrich its substrate spectrum with melibiose, Boucher et al. [90] cloned and expressed in *Lc. lactis* the α-galactosidase gene (*aga*) and its putative transcription regulator (*galR*) from *Lc. raffinolactis* ATCC 43920. The authors further improved the construct by introducing a galactose permease gene (*galA*) and a phage defense mechanism [90]. The *melA* gene from *Limosilactobacillus fermentum* CRL72 encodes a thermostable and relatively pH-resistant α-galactosidase with possible applications in the removal of α-GOS from soy products or, if it is cloned into probiotic strains, the degradation of α-GOS in situ in the gut [91]. Similar α-galactosidases have been isolated from *L. curvatus* R08 and *Leuconostoc mesenteroides* JK55 [92]; a homodimeric α-galactosidase from *L. helveticus* ATCC 10797 showed the remarkable ability to hydrolyze “flatulence sugars” such as raffinose, stachyose, and melibiose within 48 h [93].

*B. longum* ssp. *longum* JCM7052 yielded a GH36 α-galactosidase, BlAg3, an 80 kDa protein of 716 amino acids with the ability to cleave the α-(1→3) bond and release galactose from α-D-Gal-(1→3)-L-Ara, a disaccharide obtained from gum Arabic (arabinogalactan used as food additive) via treatment with another enzyme, a 3-O-α-D-galactosyl-α-L-arabinofuranosidase (GAfase), isolated from the same strain. From non-pretreated gum Arabic, however, BlAg3 was not able to hydrolyze the galactose moiety, although, for the Gal-Ara disaccharide, it showed 2- and 4-times higher affinity than it did for melibiose and raffinose, respectively. This might provide some insight into the mechanism by which gum Arabic exerts its probiotic effect and stimulates the growth of bifidobacteria [94]. Some bifidobacteria, such as *B. breve* UCC2003, have the necessary α-galactosidases to utilize even melezitose, the trisaccharide (α-D-Glc-(1→3)-ꞵ-D-Fru-(2→1)-α-D-Glc) found in honeydew and manna. This ability was due to the *mel* gene cluster (*melA-E*), which is adjacent to that for raffinose (*raf*) but much rarer in *Bifidobacterium* spp.—it was also found in two strains of *B. longum* (KACC 91653 and 55813), but not in any other *B. breve* (CECT 7263, ACS-071-V-Sch8b, DSM 20213) or *B. longum* (NCC 2705, ATCC 15697) strain, nor *B. dentium*, *B. adolescentis*, or *B. animalis* [95].

*Bacillus coagulans*, a thermophilic probiotic, is the source of α-galactosidase with remarkable resistance to various proteases (proteinase K, subtilisin A, α-chymotrypsin, and trypsin) and high hydrolytic activity able to degrade completely melibiose, raffinose and stachyose (5 g/L each) in less than 30 min. The enzyme is inhibited by 100 mM galactose, but its activity is reduced by only 25% [96]. A proteomic analysis of *Lpl. plantarum* P-8, engineered for higher utilization of raffinose, revealed that a total of 125 and 106 proteins were significantly (>1.5-fold, *p* < 0.05) upregulated and downregulated, respectively, when the strain was grown on a medium with raffinose [97].

Manninotriose, the trisaccharide Gal-α(1→6)-Gal-α(1→6)-Glc produced by MelA, a cold-active and stereospecific α-galactosidase with a strong preference for α-(1→6) linkages found in *Lpl. plantarum* WCFS1 [98], demonstrated the prebiotic ability to stimulate the growth of probiotic strains like *Lpl. plantarum* WCFS1 and *B. adolescentis* DSM 20083 [99].

Many *Lactobacillus* spp., which are isolated from foods, possess α-transgalactosylase activity. For instance, the α-galactosidase AglB from *L. amylolyticus* L6, from the naturally fermented tofu whey, was able to hydrolyze raffinose and stachyose but also to synthesize α-GOS with DP3–DP5 utilizing high-concentrated melibiose (300 mM). However, no serious attempt was made to characterize these GOS regarding the monomer units and linkage types, much less to test their probiotic potential [100]. Some data from a murine model show that α-GOS are more potent prebiotics than ꞵ-GOS and might even provide a more effective therapy for ulcerative colitis [101]. Another study in mice used a mixture of α-(1→3)-GOS derived from fungal hydrolysis and found out that after one week of treatment, the GOS-supplemented animals had higher numbers of *Prevotella* and decreased abundance of *Escherichia*, an indication of promising probiotic potential [102].

Fungi, in general, are less potent sources of α-galactosidases than bacteria. *Penicillium oxalicum* SO contained an α-galactosidase with very low hydrolyzing activity and a high rate of transgalactosylation. Using melibiose as a substrate and 6-α-galactosyl melibiose as a significant product, the enzyme achieved a transfer ratio of 83.6%, which was maintained for 80 h [103]. An α-galactosidase from *Aspergillus* sp. MK14 could hydrolyze guar gum, a galactomannan polysaccharide extracted from guar beans, and use it as a donor (instead of the more expensive melibiose or *p*NPG) for the galactosylation of glycerol. Galactosyl glycerol is an essential precursor in synthesizing functional glycolipids [104]. *Streptomyces* sp. HJG4 and *Mesorhizobium* JB07 also yielded some peculiar α-galactosidases, capable of being activated by zinc and lead (common inhibitors) or tolerating an alkaline environment (optimal pH 8) [105].

### 4.2. β-Galactosidases of Probiotic Bacteria

#### 4.2.1. Multimeric Organization, Active Site, Influence of Cations

Structural diversity is characteristic of bacterial β-galactosidases and is responsible for the equally wide range of substrates they can utilize. The classic LacZ β-galactosidase of *E. coli* is a homotetramer comprised of four identical polypeptide chains, 1023 amino acids each, and is able to hydrolyze many galactose-containing substrates [106]. The non-galactose aglycon moiety could even be *ortho*- or *para*-nitrophenol, comprising the compounds ONPG and PNPG, respectively, which are the two most frequently used substrates in enzyme assays for galactosidase activity [107]. β-Galactosidase of *E. coli* contains an active site that alternates between the “shallow” and “deep” modes when the substrate binds. Monovalent metal cations, notably Na^+^ and K^+^, bind specific amino acid residues in the active site and are crucial for stabilizing the substrate and the transition state [108].

Mg^2+^ is one of the most consistent activators of the β-galactosidase. Studies with mutant variants of the enzyme revealed that Glu-416, His-418, and Glu-461 are the prime binding sites for Mg^2+^. The catalytic activity diminishes if these amino acids are changed, and the optimum pH significantly alters [109]. Moreover, there are some cases where the apparent activation by other divalent metal cations (Ca^2+^) may be due to Mg^2+^ impurities. Extensive studies with allolactose trapped in the active site have elucidated much about the formation, conformation, and stabilization of the transition state and even extended the knowledge about *lac* operon evolution [110,111].

However, there are cases in which the influence of metal ions on enzyme activity is entirely different. A novel β-galactosidase (lacZBa) from *B. aryabhattai* GEL-09, cloned and expressed in *E. coli*, proved to be activated by Mn^2+^, Zn^2+^, Fe^2+^, Mg^2+^, Ca^2+,^ and, most intensely and most surprisingly, by Co^2+^ [112]. Another β-galactosidase from *Aspergillus oryzae*, immobilized on concanavalin A-modified silica-coated titanium dioxide nanocomposite, showed enhanced activity by metal cations in the order Mg^2+^ > K^+^ > Na^+^ > Ca^2+^ [113]. The same study also found that activation was dose-dependent for Na^+^ and Ca^2+^ between 20 and 25 mM, and for Mg^2+^ and K^+^, it was between 40 and 45 mM, allowing enzyme activity to be fine-tuned. Some β-galactosidases from thermophilic bacteria, such as *Alicyclobacillus vulcanalis* DSM 16176, are not affected by the presence of heavy metal ions such as Cd^2+^, Co^2+^, Fe^2+^, Fe^3+^, Hg^2+^, and Ni^2+^, none of which reduced enzyme activity by more than 20%, and Cu^2+^ had no effect at all [114,115]. A description of the characterized β-galactosidases from probiotic bacterial strains is shown in Table 4.

**Figure 4:**
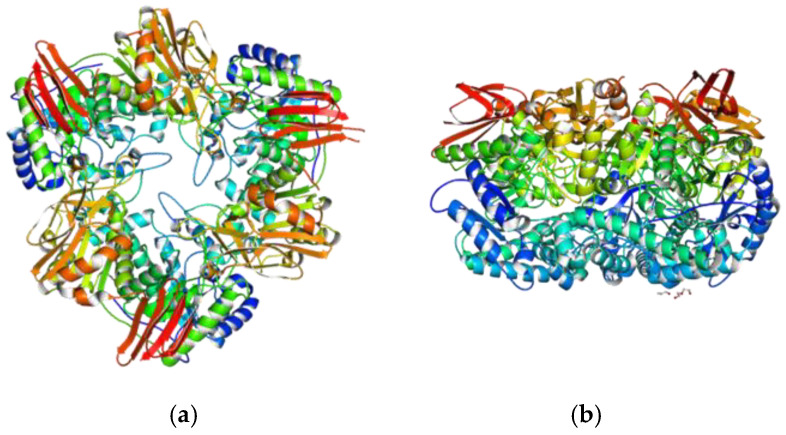
(**a**) Three-dimensional model of *B. bifidum* β-galactosidase (trimer) made in the SWISS-MODEL Workspace [85]; (**b**) the same model, rotated by 90° (horizontally).

In *B. longum* ssp. *infantis* and *B. bifidum* S17 [127,128], β-galactosidase acts as a homotrimer (Figure 4).

The β-galactosidases of the extremophiles *Thermus thermophilus* A4 [129] and *Picrophilus torridus* DSM 9790 are homotrimers [114], and the enzyme in the psychrophilic bacterium *Marinomonas* ef1 is a hexamer [130].

The heterodimeric structure is typical for β-galactosidases from the *Lactobacillaceae* family. The enzymes usually consist of small (35 kDa) and large (72 kDa) subunits encoded by two adjacent genes, *lacL* and *lacM*, both confirmed in *L. reuteri* L461 [123], *Lpl. plantarum* WCFS1 [124], *L. acidophilus* R22 [117], and *Lim. fermentum* K4 [118]. Most β-galactosidases in the *Lactobacillaceae* family have similar pH and temperature optimums: a pH between 6.5 and 7.0 and a temperature of 55–60 °C. Likewise, identical metal cations act as enzyme activators (K^+^, Na^+^, Mg^2+^, Mn^2+^) and inhibitors (Ca^2+^, Zn^2+^, Cu^2+^) [121]. Similar optimal conditions were reported for the homotetrameric enzyme of *L. bulgaricus* 43, with atypical Ca^2+^ ions acting as activators [116].

#### 4.2.2. Catalytic Mechanism and Linkage Preference

The enzymatic reactions of hydrolysis and transglycosylation performed by β-galactosidase follow the two-step mechanism described above, but the two processes differ in the type of acceptor (nucleophile) destroying the acyl-enzyme complex. Amino acid substitutions of the catalytic nucleophile are the most effective way to abolish the hydrolytic activity. Since this event also affects the transglycosylation, glycosyl donors that mimic the glycosyl-enzyme complex (e.g., glycosyl fluorides) may be used to enhance GOS synthesis [131].

In the transglycosylation process, *E. coli* β-galactosidase predominantly forms the disaccharides lactose and allolactose with β-(1,4) and β-(1,6) linkages, which are thermodynamically favorable. That is why allolactose is the natural inducer of the *lac* operon in *E. coli* and is therefore required for the expression of β-galactosidase [132].

β-Galactosidases differ greatly in their transgalactosylation activity and produce GOS of varying lengths and linkages. *L. helveticus* DSM 20075, *L. reuteri*, *L. bulgaricus*, *S. thermophilus*, and *B. breve* have demonstrated a marked preference for GOS with β-(1→6) and β-(1→3) bonds. While β-(1→4) bonds were also detected, they remained firmly in the minority [133]. In contrast, a β-galactosidase from *L. delbrueckii* ssp. *bulgaricus* was able to synthesize DP3 GOS with the atypical β-(1→4) as the most abundant type of linkage, with a slight excess of the β-(1→3) bond [26,116]. Another β-galactosidase isolated from *L. bulgaricus* (strain L3) produces two trisaccharide isomers, one with β-(1→3) and one with the β-(1→4) bond between the two galactosyl monomers [134,135].

Studies with site-directed mutagenesis of the β-galactosidase BgaD and its C-terminally truncated variant BgaD-D from *B. circulans* ATCC 31382 have provided considerable insights into the catalytic mechanism and the linkage preference in the final GOS. Site-directed mutants Glu447Asn, Glu532Gln, His345Phe, and His379Phe lost all or nearly all catalytic activity. Glu447 was identified as the general acid/base catalyst, Glu532 as the nucleophile, and His345 and His379 as stabilizers of the transition state [136]. Site-saturation mutagenesis of two Arg residues proved crucial for the produced GOS content. The Arg484Ser and Arg484His mutants produced 14 structures that were not present in the GOS mixture of the wild-type enzyme. The new GOS contained a markedly increased number of β-(1→3) bonds and a 50 times greater amount of the trisaccharide β-Gal-(1→3)-β-Gal-(1→4)-Glc [137]. Two other residues in the active site, Asp481 and Lys487, have been identified as responsible for linkage preference. Changes like Asp481Glu, Asp481Asn, Lys487Ser, and Lys487Gly dramatically increased the production of a trisaccharide with the β-(1→3) bond between the galactosyl moieties, while the synthesis of all other GOS decreased [138].

Experiments with ^13^C-labeled glucose and galactose showed that the β-galactosidases of *B. circulans*, *Kluyveromyces lactis*, and *A. oryzae* yielded GOS with different structures. The enzyme from *B. circulans* possessed the highest activity and formed complex tri- and tetrasaccharides with β-(1→2/3/6) bonds. It uses free glucose as an acceptor rather than glucose retained in the active site. The *A. oryzae* and *K. lactis* enzymes preferred the β-(1→3) bond. However, β-galactosidase of *A. oryzae* synthesized GOS with β-(1→4) linkages, while that of *K. lactis* does not [139].

## 5. Trends in GOS Production Enhancement

Biotechnologies for GOS synthesis engage whole microbial cells or enzymes such as natural, engineered, free, or immobilized α- and β-galactosidases. The immobilization methods include encapsulating the enzyme in carriers and covalent binding [42]. The most widely used galactosidases in industry are fungal. Kote et al. [140] focused on the high-yield production and detailed biochemical characterization of the α-galactosidase enzymes derived from the *Penicillium* species. Culture conditions optimization, including temperature, pH, and nutrient composition, have been done to enhance the enzyme yields. The biochemical analysis revealed that the enzyme performed optimally within specific pH and temperature ranges, demonstrating stability and efficiency.

In the food industry, large quantities of α-galactosidase are used to treat legumes high in sugars, causing flatulence. This enzyme is also needed in biofuel production for the hydrolysis of plant materials. *Saccharomyces cerevisiae* α-galactosidase appeared highly efficient in breaking down RFO, including raffinose, stachyose, and verbascose—all compounds that cause human digestive issues [141].

Considerable scientific efforts have been directed towards improving the processes of α-galactosidase production by one factor per time (OFT) optimization. However, the Central Composite Design (CCD) and the Response Surface Methodology are much more efficient methods, as demonstrated by their application to the production of extracellular α-galactosidase by *A. niger* NRC114 [142] and in solid-state fermentation for extracellular enzymes produced by *Fusarium moniliforme* NCIM 1099 [143].

Bacterial β-Gal is usually preferred in industrial applications for its high activity and stability, high enzyme yields, and rapid growth rates of the producing strains [144]. LABs are recommended sources of enzymes in the food industry due to their health benefits and GRAS status [124]. However, they are generally mesophilic; therefore, strain engineering, encapsulation, or specific media can increase their tolerance to heat stress to enhance their thermostability. The potential to modify β-Gal-producing microorganisms is crucial for the cost-effective and efficient use of industrial processes involving this enzyme. The preferred medium for producing β-Gal by LAB consisted of whey, skim milk, or whey permeate, enhanced with either 1.0% whey protein, 0.2% yeast extract, or 1.2% MRS broth. *Lactobacillus delbrueckii* ssp. *bulgaricus* CRL450 is a strain with an exceptionally high β-gal activity of 2.06 U/mg and high GOS-producing ability. It formed 41.3% GOS with predominant β-(1→6) bonds in 5 h [31]. The β-galactosidase gene from *L. bulgaricus* L3 was cloned and expressed in *E. coli* using the pET-21b vector. The purified recombinant enzyme could transfer glycosyl groups from lactose to sucralose. The main product reached 41.0% GOS at a lactose/sucralose ratio of 1.5/1 after 15 h [145]. *L. reuteri* CF2-7F, grown on lactose, showed the highest activity of β-galactosidase (82.01 U/mL), and yeast extract is the best protein source to achieve a high amount of β-Gal [146]. The *lacZ* genes of *L. bulgaricus* strains 1.1480 and wch9901 were found to differ, leading to variations in theβ-galactosidase production properties. Given its high β-galactosidase activity, strain wch9901 appears to be a promising candidate as a parental strain for developing a food-grade β-galactosidase delivery system [147]. According to another study, the recombinant β-Gal enzyme from *Lpl. plantarum* WCFS1 was over-expressed within the same species. This enzyme owns high transgalactosylation activity, leading to high yields of GOS with prebiotic properties. A maximum GOS of 41% (*w*/*w*) was achieved at an 85% lactose conversion using an initial lactose concentration of 600 mM. The enzyme predominantly formed β-(1→6) linkages during transgalactosylation, with β-(1→3) linkages occurring less frequently. The primary GOS products were β-D-Galp-(1→6)-D-Lac, which accounted for 34% of the total GOS, and β-D-Galp-(1→6)-D-Glc, which comprised 29% of the total GOS [124]. The β-galactosidase enzyme from the promising probiotic strain *B. longum* BCRC 15708 synthesized GOS through transgalactosylation, using lactose as the substrate. The reaction primarily produced two types of galactans: trisaccharides and tetrasaccharides, with the trisaccharides being the predominant product. The highest amount of GOS was 32.5% (*w*/*w*) from the 40% lactose solution, as the lactose conversion reached 59.4% [148].

The most widely used yeasts in the industry for β-Gal enzyme production are *K. marxianus* and *K. lactis. K. marxianus* is more attractive for industrial applications due to its GRAS status, high production rate, greater catalytic efficiency, and enzyme stability. While yeasts generate intracellular β-Gal, fungi such as *A. niger* and *A. oryzae* produce extracellular enzymes [139]. Intracellular β-Gal is more tolerable at a pH range of 6.5–7 and a temperature optimum of 50–60 °C and is commonly used for the hydrolysis of lactose in milk or whey. On the other hand, extracellular β-Gal has a pH optimum between pH 3 and 5 and a temperature optimum of 30–35 °C, as this cold-active enzyme allows the treatment of milk and dairy products under mild conditions with the unique taste and nutritional values remaining unchanged. The fungal β-gal enzyme is thermostable and is preferably used in industry to minimize microbial contamination [149].

In *Lactobacillaceae*, GOS yields typically reach 20–40% conversion of the initial lactose before the hydrolytic activity of the β-galactosidase becomes preferable on purely kinetic grounds [127]. Exceptional cases with GOS yields close to 50% at 400 g/L (1.17 M) initial lactose have been achieved by various techniques. Permeabilized whole cells of *L. lactis* LM0230, with lacS from hyperthermophile *Sulfolobus solfataricus* successfully expressed, achieved 197 g/L GOS tri- and tetrasaccharides after 55 h incubation at 85 °C, or slightly more than 49% lactose conversion [150]. The β-galactosidase gene of *L. bulgaricus* L3 (*bgaL3*) was fused with the cellulose-binding domain (CBD), expressed in *E. coli*, and adsorbed on microcrystalline cellulose. The immobilized fusion enzyme reached a GOS yield of 49% at 400 g/L lactose within a mere 75 min at 45 °C [151]. Cloning of the *lacZ* gene from *L. delbrueckii* ssp. *bulgaricus* DSM 20081 and its expression in *Lpl. plantarum* WCFS1 reached an almost 50% conversion of lactose, but only at a 205 g/L (600 mM) initial concentration [152].

Immobilization has been a popular method to improve various properties of β-galactosidases, especially their stability. Covalent immobilization on glyoxyl-agarose of β-galactosidase from *Lpl. plantarum* led to a more than 20-fold increased stability compared to the soluble enzyme and even one new trisaccharide product from lactulose [125]. Immobilization, however, seldom leads to increased enzyme activity or efficiency, and even the increased stability may come at the cost of tedious optimization [153]. Bayramoglu et al. [154] immobilized a β-galactosidase from *A. oryzae* via covalent binding onto double-layered hydrophilic polymer-coated magnetic nanoparticles and thus increased its optimum temperature by 15 °C. However, *Km* increased by 18%, and *Vmax* decreased by 28%. β-Galactosidase from *Lpl. plantarum* HF571129, immobilized by adsorption and cross-linking on ZnO nanoparticles, demonstrated greatly improved thermal stability, with the altered enzyme becoming almost thermophilic and shifting its temperature optimum by nearly 10 °C towards higher temperatures. Still, the pH range was slightly broadened, and the storage life was improved by a mere 16% [155]. Immobilization of whole cells may be a more prospective method. The β-galactosidase activity of *S. lactis* showed a most impressive improvement after entrapment in silica microcapsules: *Km* decreased by 50% (from 8.33 mM to 4.16 mM), and *Vmax* increased by nearly 75% (from 71.43 to 125 μmoL/min) [156].

New omics approaches and genetic engineering techniques can further be engaged in studying the regulation of processes and developing more efficient recombinant enzymes. GOS utilization genes are widely distributed in *Bifidobacterium* species (*B. longum*, *B. breve*, *B. bifidum*) and conserved in adult- and infant-type strains [157]. Differential transcriptomics in the *B. breve* strain YIT 4014T revealed that GOS utilization genes are organized in clusters which include, in addition to the obligatory GH32 family β-galactosidase, several genes for an ABC transporter system and a transcriptional regulator from the LacI family [158]. A transcriptomic study in *L. acidophilus* NCFM identified several GOS-induced genes within the 16.6-kb gal-lac cluster. Most notable among them are two genes for β-galactosidases (*lacA* and *lacLM*) and one encoding lactose permease (*lacS*), all of which were upregulated between 4.8 and 6.2 times in the presence of GOS [159]. The *gal* locus of *B. breve* UCC2003 has provided further insight into GOS utilization. The locus contains GOS-inducible genes encoding a β-1,4-endoglucanase (*galA*), an extracellular enzyme responsible for the generation of shorter GOS, a transport system (*galBCDE*), and a β-galactosidase from the GH42 family (*galG*) that further hydrolyses them to galactose monomers [160,161]. Therefore, engineered strains may be employed to improve GOS production. For example, site-directed and site-saturated mutagenesis of the β-galactosidase BgaB from *Geobacillus stearothermophilus* KVE39 yielded several mutants with considerably higher GOS-producing capacity. Arginine at position 109 was exchanged for lysine, valine, and tryptophan, and the obtained mutants produced 11.5%, 21%, and 23.5% (*w*/*w*) of the trisaccharide 3′-galactosyl lactose compared to only 2% for the wild-type enzyme [162]. Site-saturation mutagenesis of one tryptophan residue (W908) in a β-galactosidase from *L. bulgaricus* L3 produced a mutant with phenylalanine instead (W980F), which was capable of increased glycosylation of phenolic compounds from 7.6% to 53.1% in the cases of phenol, hydroquinone, and catechol. The mutant also generated 32.3% glycosides from pyrogallol, a compound that could not be glycosylated by the wild-type enzyme [163].

## 6. Conclusions

Galactose-containing oligosaccharides (α- and β-GOS) fully meet the criteria for a prebiotic carbohydrate: they are not degraded by human enzymes, promote the multiplication of beneficial microbiota in the GIT, and display well-documented positive effects on human organisms, such as supporting the digestive, immune, and nervous systems, and alleviating symptoms of allergies and autism. Prebiotic strains from the genera *Bifidobacterium* and *Lactobacillus* possess specific enzyme systems for the degradation of GOS, transport into the cell, and utilization of the resulting monosaccharides, but also the ability to synthesize GOS of different types due to the combined transferase and hydrolase activity of α- and β-galactosidases. These enzymes are multimeric, in most cases trimers or tetramers, have several active centers, and are inductive, i.e., initiation of transcription of the responsible operons occurs in the presence of GOS.

Since GOS have proven prebiotic properties, interest in their industrial production has grown enormously over the last decade. Therefore, in addition to traditional sources of galactosidase enzymes, such as lactic acid bacteria, *B. coagulans*, bifidobacteria, and fungi (*A. oryzae*, *A. niger*), the search for more potent GOS producers and highly active enzymes includes engineered producers such as *E. coli* and yeast. However, the mechanisms of the delicate interaction between probiotic bacterial strains and prebiotic carbohydrates are not entirely clear. Therefore, future in-depth analyses of their relationships at the transcriptomic and proteomic levels are forthcoming.

## Figures and Tables

**Table 1:** Chemical structure of α-GOS. The structural formulas were obtained from the free database PubChem (NCBI) [40].

Name	Formula IUPAC (Condensed)	2D Structure
Raffinose	Gal(a1→6)Glc(a1→2b)Fru	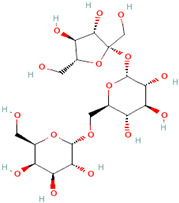
Stachyose	Gal(a1→6)Gal(a1→6)Glc(a1→2b)Fru	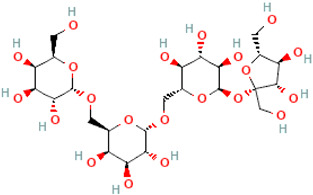
Verbascose	Gal(a1→6)Gal(a1→6)Gal(a1→6)Glc(a1→2b)Fru	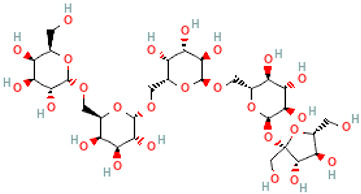
Ajugose	Gal(a1→6)Gal(a1→6)Gal(a1→6)Gal(a1→6)Glc(a1→2b)Fru	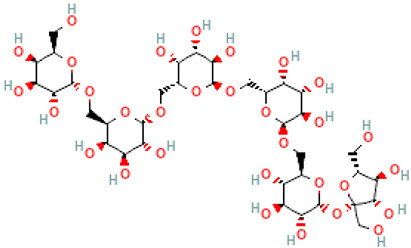
Lychnose	α-D-Gal-(1→1)-β-D-Fru-(2↔1)-α-D-Glc-(6←1)-α-D-Gal (Gal-1-6-Glc-1-2-Fru-1-1-Gal)	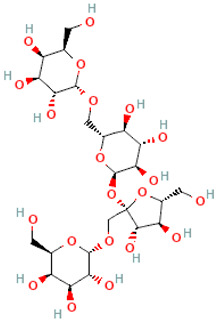
Isolychnose	α-D-Gal-(1→3)-β-D-Fru-(2↔1)-α-D-Glc-(6←1)-α-D-Gal (3F-α-D-Galactosylraffinose)	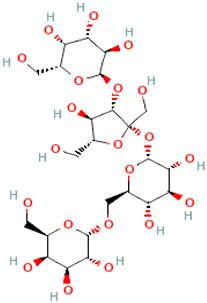
Stellariose	α-D-Gal-(1→1)-β-D-Fru-(2↔1)-α-D-Glc-[(4←1)-α-D-Gal-(6←1)-α-D-Gal	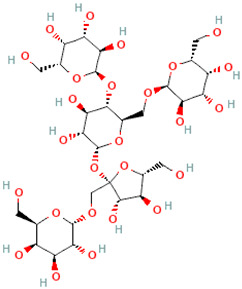

**Table 2:** Structures of β-GOS. The structural formulas were obtained from the free database PubChem [40].

Name	Formula IUPAC (Condensed)	2D Structure
3-Galactosyllactose	Gal(αl→3)-Gal(βl→4)Glc	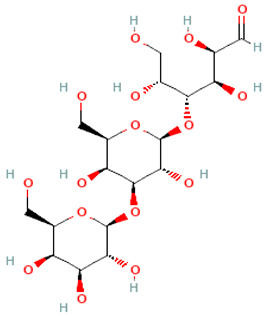
4-Galactosyllactose	β-Gal-(1→4)-β-Gal-(1→4)-Glc	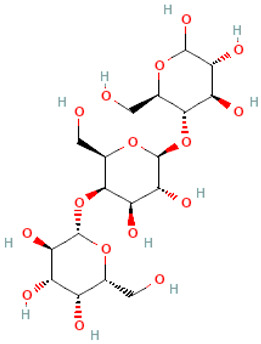

**Table 3:** Purified and characterized α-galactosidases of probiotic bacteria.

Species, Strain	Enzyme	pH/ T (°C)	Metal Ions	Substrate	GOS	Reference
*B. lactis* B94	Purified	37 °C	ND	Soymilk	α-(1-6)	[64]
*S. thermophilus* St1342	Purified	37 °C	ND	Soymilk	α-(1-6)	[64]
*L. acidophilus* La4962	Purified	37 °C	ND	Soymilk	α-(1-6)	[64]
*B. adolescentis* DSM 20083	344 kDa (79 kDa monomer)	pH 5.5, 55 °C	ND	Raffinose, stachyose	α-(1-6), α-(1-3), α-(1-4) De novo GOS synthesis	[75]
*B. breve* DSM 20213	160 kDa, (80 kDa monomer)	pH 5.5, 37 °C	↓ Cu^2+^, Hg^2+^	Raffinose, melibiose	α-(1-6), α-(1-3), α-(1-4)	[64]
*B. breve* 203	Aga2, 81.5 kDa monomer	pH 5.5, 50 °C	↑(NH)_4_^+^, EDTA ↓ Cu^2+^, Hg^2+^, Ag^+^	Melibiose	De novo synthesis of a trisaccharide [Gal-α-1, 4-Gal-α-1, 6-Glc]	[76]
*B. longum*, *B. pseudocatenulatum*	Purified (2 enzymes)	pH 6.0, 40 °C	↑ Zn^2+^, Na^+^, Ca^2+^, Mn^2+^ ↓ Al^3+^	Raffinose	GOS of α-D-galactose + L-arabinose, α-D-galactose + sucrose	[64]
*B. bifidum* NCIMB 41171	MelA, 243 kDa, 85 kDa monomer	pH 6.0	ND	Melibiose	Synthesis of GOS with DP ≥ 3, the total yield of 20.5% (*w*/*w*)	[77]

**Table 4:** Purified and characterized β-galactosidases of probiotic bacteria.

Species, Strain	Mw (kDa)	3D Structure	pH, T (°C)	Metal Ions	Substrate	β-GOS	Reference
*L. bulgaricus* 43	150	Tetramer	pH 6.5 55 °C	↑ Mn^2+^, Mg^2+^, Ca^2+^ ↓ Zn^2+^, Cu^2+^	Lactose	DP3, DP4	[116]
*L. acidophilus* R22	107	Heterodimer	pH 6.5 55 °C	↑ Mg^2+^ ↓ Mn^2+^, Cu^2+^, Zn^2+^	Lactose	DP2	[117]
*Lim. fermentum* K4	107	Heterodimer	pH 6.5–7.0 40–50 °C	↑ Na^+^, K^+^, Mg^2+^	Lactose	ND	[118]
*L. helveticus* DSM 20075	110	Heterodimer	pH 6.5 55–60 °C	↑ K^+^, Na^+^ ↓ Mn^2+^, Mg^2+^, Ca^2+^, Zn^2+^	Lactose, ONPG ^1^	DP2, DP3, DP4	[119]
*L. curiae* M2011381	ND	Heterodimer	pH 8.0 55 °C	ND	Lactose	ND	[120]
*L. acidophilus* MR-24	110	Heterodimer	pH 7.0 37 °C	↑ Mg^2+^, Ca^2+^ ↓ Zn^2+^, Cu^2+^	Lactose	ND	[121]
*B. longum* ssp. *infantis* BiBga42A	73.5	Trimer	pH 7.0 45 °C	ND	Lacto-N-tetraose	ND	[122]
*L. reuteri* L103	105	Heterodimer	pH 6.0 50 °C	↑ Na^+^, K^+^, Mn^2+^	ONPG ^1^, Lactose	ND	[123]
*L. reuteri* L461	105	Heterodimer	pH 6.5 55 °C	↑ Na^+^, K^+^, Mn^2+^	ONPG ^1^	ND	[123]
*L. mucosae* OLL 2848	ND	ND	37 °C	ND	ONPG ^1^	ND	[67]
*Lpl. plantarum* WCFS1	107	Heterodimer	pH 6.5–8.0 30 °C	↑ Mg^2+^, Mn^2+^	ABTG ^2^	DP3, DP2	[124]
*Lpl. plantarum*	ND	ND	pH 6.5 50 °C	↑ Mg^2+^, Ca^2+^ ↓ carbohydrates	Lactose, Lactulose	DP3, DP4	[125]
*L. crispatus* ATCC 33820	ND	ND	pH 6.5 50 °C	↑ Fe^2+^, Mn^2+^ ↓ Zn^2+^	Lactose	ND	[126]

^1^ ONPG, *ortho*-Nitrophenyl-β-galactoside; ^2^ ABTG, *para*-Aminobenzyl-1-thio-β-D-galactopyranoside; ND, not determined.
